# A Hybrid Approach Based on Higher Order Spectra for Clinical Recognition of Seizure and Epilepsy Using Brain Activity

**DOI:** 10.29252/NIRP.BCN.8.6.479

**Published:** 2017

**Authors:** Seyyed Abed Hosseini

**Affiliations:** 1. Research Center of Biomedical Engineering, Mashhad Branch, Islamic Azad University, Mashhad, Iran.

**Keywords:** Epilepsy, Electroencephalogram, Higher order spectra, Seizure

## Abstract

**Introduction:**

This paper proposes a reliable and efficient technique to recognize different epilepsy states, including healthy, interictal, and ictal states, using Electroencephalogram (EEG) signals.

**Methods:**

The proposed approach consists of pre-processing, feature extraction by higher order spectra, feature normalization, feature selection by genetic algorithm and ranking method, and classification by support vector machine with Gaussian and polynomial radial basis function kernels. The proposed approach is validated on a public benchmark dataset to compare it with previous studies.

**Results:**

The results indicate that the combined use of above elements can effectively decipher the cognitive process of epilepsy and seizure recognition. There are several bispectrum and bicoherence peaks at every bi-frequency plane, which reveal the location of the quadratic phase coupling. The proposed approach can reach, in almost all of the experiments, up to 100% performance in terms of sensitivity, specificity, and accuracy.

**Conclusion:**

Comparing between the obtained results and previous approaches approves the effectiveness of the proposed approach for seizure and epilepsy recognition.

## 1. Introduction

Epilepsy is a common nervous system disorder characterized by sudden and recurrent seizures (Chisci et al., 2010). The World Health Organization (2017) statistics show that approximately 50 million people worldwide currently suffer from epilepsy. Epileptic seizures are categorized into focal (also called partial or localization-related), generalized, and unclassified. In focal seizures, the abnormal electrical discharges start with a localized region, whereas in the generalized seizures, the abnormal electrical discharges start in both hemispheres of the brain simultaneously (Dekker, 2002). Generalized seizures are usually divided into several main types, including absence typical (also known as petit mal), absence atypical, myoclonic, tonic, clonic, tonic-clonic (also known as grand mal), and atonic (also known as astatic). Focal seizures are also divided into three main types; simple, complex, and secondarily generalized seizures (Engel, 2006).

The recognition of epilepsy is usually achieved by visual viewing of Electroencephalogram (EEG) recordings, by an experienced neurologist or neurophysiologist. However, this approach is very time-consuming, especially in the case for long-term EEG recordings that may even last for several days. As a solution, analysis of brain signals such as EEG and Electrocorticogram (ECoG) have been used to recognize different epilepsy states. Brain signals are usually recorded in two essential ways; 1. Non-invasive recording, which is known as scalp EEG recordings; and 2. Invasive recording, which is often known as intra-cranial EEG (ECoG signal).

Several research studies have been undertaken in epilepsy recognition over the last few years (Berkovic & Crompton, 2010). They are usually classified into four major categories; including diagnosis, prediction, localization, and recognition (Berkovic & Crompton, 2010). In most of the studies, choosing appropriate features is one of the most challenging task. Therefore, many features have been investigated based on wavelet transformation analysis (Subasi, 2007; Güler & Übeyli, 2004; Ocak, 2009), Time-Frequency Analysis (TFA) (Srinivasan, Eswaran, & Sriraam, 2007; Nigam & Graupe 2004), Fourier transformation analysis (Ktonas, 1987), energy distribution in time-frequency plane (Tzallas, Tsipouras, & Fotiadis, 2007; Tzallas, Tsipouras, & Fotiadis, 2009), Higher Order Spectra (HOS) analysis (Hosseini, Khalilzadeh, Naghibi Sistani, & Niazmand, 2010; Chandran, Acharya, & Lim, 2007), and chaos theory based analysis (Ocak, 2009; Srinivasan, Eswaran, & Sriraam, 2007). Feature selection, classification, offline and online processing in recognition of different epilepsy states are other challenging issues.

The spectral analysis is a powerful tool for reconstruction of process properties from measured data (Hosseini et al., 2010; Hosseini, 2009). In the meantime, HOS analysis is a well-established signal analysis technique in communication with many applications in science (Hosseini et al., 2010; Hosseini, 2009; Abootalebi, 2000; Xiang & Tso, 2002; Shahid, Walker, Lyons, Byrne, & Nene, 2005). In this paper, after pre-processing, HOS features such as bispectrum, bicoherence, and Hinich’s test are extracted from brain signals with both quantitative and qualitative perspectives. Then, a Genetic Algorithm (GA) is used to select optimum features in order to recognize different epilepsy states. The analysis further confirms through the statistical analysis with value less than 0.001 for the extracting best feature. Best features are used with a Support Vector Machine (SVM) with Gaussian and polynomial Radial Basis Function (RBF) kernels in order to recognize different epilepsy states. The main contribution of the present study is proposing a more reliable and efficient clinical technique based on HOS to classify different epilepsy states, including healthy, interictal, and ictal states, by EEG signals.

This paper is organized as follows: The previous studies are presented in this section. The methods and materials are given in Section 2. The experimental results are illustrated in Section 3. The discussions are illustrated in Section 4.

This section presents a detailed discussion of previous related studies on feature extraction using linear and nonlinear methods along with different machine-learning classifiers. To date, several methods have been proposed for recognizing of different epilepsy states using brain signals. Gotman (Gotman, 1982) was one of the first researchers who recognized epileptic events in EEG signals and presented a method for seizure recognition. Murro et al. (1991) proposed a seizure recognition approach based on three features, including dominant frequency, relative amplitude, and rhythmicity of the ECoG signal. They achieved a recognition sensitivity of 91%–100%. Liang, Wang, and Chang (2010) designed a combination of complexity and spectrum analysis for recognition of different epilepsy states. They used Principal Component Analysis (PCA) and GA as two feature selection methods, which PCA provided better results than GA.

Lima and Coelho (2011) used an SVM with different kernels, including the standard, least squares, Lagrangian, proximal, smooth, and relevance for epilepsy recognition. They concluded that all the mentioned kernels are in a competition in terms of accuracy. Tzallas, Tsipouras, and Fotiadis (2009) presented a TFA for detection of epileptic seizure. They used statistical analysis between the achieved accuracy from the Reduced Interference Distribution (RID) and the Short-Time Fourier Transform (STFT) for all classification problems. Their method can distinguish between healthy and ictal state up to 100% accuracy. Musselman and Djurdjanovic (2012) presented TFA for epilepsy recognition. Their results are able to outperform the accuracy of the previous research for epilepsy recognition.

Alam and Bhuiyan (2013) provided a technique using statistical features, including variance, kurtosis, and skewness, for epilepsy recognition. Their method is faster in comparison with the TFA. Tezel and Ozbay (2009) proposed a method based on three types of Neural Networks (NNs) with adaptive activation function, including “Morlet wavelet function”, “sigmoid function”, and “sum of sigmoid and sinusoidal function” with free parameters for epileptic seizure detection. They achieved approximately 100% sensitivity, specificity, and accuracy in all experiments. Karayiannis et al. (2006) proposed a seizure detection in the neonatal EEG signal using a rule-based method cascaded with an NN. Their results indicated that the trained NNs improved the performance of the rule-based methods acting by themselves. Niknazar et al. (2013) presented two different approaches such as thresholding and classification for detection of seizures in rats using ECoG signals. Their results showed that the best results are obtained by the coastline feature that led to a two second delay in its correct detections and the fuzzy similarity index that led to a value lower than 0.001. Fathima, Khan, Bedeeuzzaman, and Farooq (2011) used a method based on Higher Order Moments (HOMs) for automatic seizure detection in healthy and ictal classes. Their approach can distinguish two different epilepsy states with 97.77% accuracy.

Rana et al. (2012) presented a seizure detection method based on the phase-slope index of direct influence, applied to multi-channel ECoG signals. Their approach detected all of the seizures in four of the five patients with a false detection rate less than two per hour using a common threshold procedure. Thomas et al. (2013) proposed a classification approach for automated neonatal seizure detection. Their approach is able to distinguish different seizure events with 75.4% accuracy. Zandi et al. (2010) presented an approach based on wavelet for real-time recognition of epileptic seizures in EEG signal. Their approach is able to obtain a high sensitivity of 90.5%. Subasi (2007) used a method based on Wavelet Coefficients (WCs) and Adaptive Neuro-Fuzzy Inference System (ANFIS) for epilepsy recognition. He concluded that the ANFIS obtained higher accuracies than the NN. Fu et al. (2015) proposed a Hilbert Marginal Spectrum (HMS) method for seizure and epilepsy detection in EEG signals. Their results indicated that the average accuracy is 99.85% for healthy versus ictal classes, and 99.8% for the entire data except ictal versus ictal classes.

To date, several chaotic approaches have been proposed for recognition of different epilepsy states. In one study, Adeli, Ghosh Dastidar, and Dadmehr (2007) presented a combination of WCs, Correlation Dimension (CD), and Largest Lyapunov Exponent (LLE) for recognition of different epilepsy states using EEG signals. Ghosh Dastidar, Adeli, and Dadmehr (2007) used a combination of WCs, Standard Deviation (SD), CD, LLE, and Levenberg-Marquardt back propagation NN for recognition of different epilepsy states using EEG signals. Their technique can distinguish the different classes, with the highest accuracy of 96.7%. López-Cuevas et al. (2013) designed an approach based on Approximate Entropy (ApEn) and recurrent NNs for recognition of high-frequency oscillations in EEG signals. Their results showed a correlation between the high-frequency oscillations and the transitions, from interictal to ictal.

More recently, Daliri (2013) proposed a kernel method based on the Earth Mover’s Distance (EMD) for epilepsy classification using EEG signals. He concluded that the kernel method is effective for epilepsy recognition. Hosseini, Akbarzadeh Totonchi, and Naghibi Sistani (2013) provided a combination of Hurst exponent (H), Petrosian Fractal Dimension (PFD), CD, LLE, and ANFIS for epilepsy recognition. Their method can be applied to both interictal and ictal classes. In another study Hosseini, Akbarzadeh Totonchi, and Naghibi Sistani (2015) proposed a correct labeling process based on PFD, H, LLE, and Bayesian classifier for epilepsy recognition. Their results showed that the minimum embedded dimension and complexity reduced in ictal state. Their technique can also distinguish the different classes, with 99.2% accuracy for healthy versus pre-ictal states, 99.7% for the healthy versus ictal states, and 97.1% for the preictal versus ictal states. In another study, Hosseini et al. (2013) proposed an approach based on H, LLE, and ANFIS for recognition of epileptic seizures. Their results indicated that the average accuracy is 97.4% for healthy versus pre-ictal states, 96.9% for the healthy versus ictal states, and 96.5% for the pre-ictal versus ictal states.

## 2. Methods

**Figure 1.**
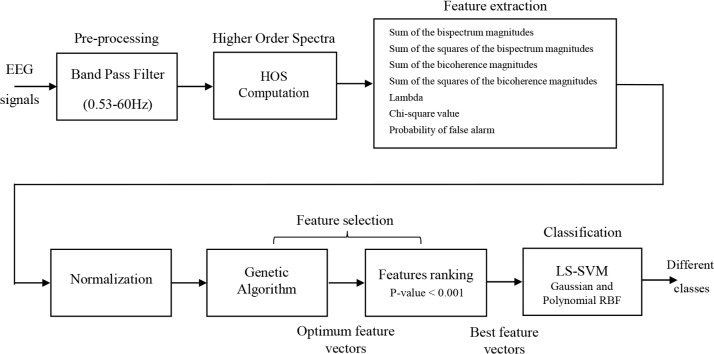
A block diagram of the proposed approach for recognition of different epilepsy sates using EEG signals

This section provides details of the clinical epilepsy database, feature extraction, feature normalization, feature selection, and classification of different epilepsy states using EEG signals. For sart, a general block diagram of the proposed approach for recognition of healthy versus ictal is shown in Figure 1. In the following, these steps are described in detail.

### 2.1. Database

The database was obtained from Bonn University, Germany (Andrzejak et al. 2001). The main reason for using the database is its widespread use in the previous research. The datasets consist of five sets (denoted A-E) that each of them contains 100-single channel EEG segments of 23.6 seconds duration. All signals were sampled at 173.61 Hz. Thus, the data point of each segment is 173.61×23.6≈4096. The data acquisition system has bandwidth between 0.5 and 85 Hz. Sets A and B have been recorded by surface electrodes from 5 healthy participants in the wake and relax states with eyes open and closed, respectively (Hosseini et al., 2013). Sets C, D and E have been recorded by depth electrodes from 5 patients in pre-surgical diagnosis (Hosseini et al., 2013). Sets C and D consist of intra-cranial EEG epochs recorded in interictal (seizure-free interval) state from the hippocampal formation of the opposite hemisphere of the brain and the epileptogenic zone of the brain (shows focal interictal activity), respectively. Set E has been recorded during seizure attack activity (ictal state).

### 2.2. Brief description of higher order spectra

The power spectrum is one of the most used feature in signal processing. The spectral moments of order larger than two are referred to as HOS (Nikias & Mendel, 1993). HOS “contain information not present in the power spectrum” (Hosseini, 2009; Xiang & Tso, 2002). As an example, traditional signal processing techniques based on the first and second order statistics are appropriate for the signals which are coming from the Gaussian and minimum phase systems, but for non-Gaussian and non-linear processes such as EEG and ECoG signals, it has lost phase information. The bispectrum is a function of two independent frequencies, f_1_ and f_2_, which could take both positive and negative values. The bispectrum is usually used due to the finite length signals and high computation and has a magnitude and a phase. Moreover, the amplitude of the bispectrum in the bi-frequency (f_1_, f_2_) plane measures the amount of coupling among the spectral components at the frequencies f_1_, f_2_, and f_1_+f_2_ (Xiang & Tso, 2002).

In real processes, discrete bispectrum has twelve symmetric regions in the bi-frequency plane (Nikias & Mendel, 1993; Swami, Mendel, & Nikias, 2003). Therefore, I extract features only in the triangular region, which include all the information of the bispectrum and bicoherence. The normalized bispectrum is called bicoherence, where the bispectrum value ranges between 0 and 1. Hinich (1982) developed methods to test for Gaussianity and linearity. More details about the Hinich’s test can be found in related studies (Hosseini, 2009; Hinich, 1982). For a more detailed description of the HOS, please refer to the relevant studies (Hosseini, 2009; Nikias & Mendel, 1993; Swami, Mendel, & Nikias, 2003).

### 2.3. Pre-processing

Before pre-processing, visual inspection is applied to all 5 sets for removing artifacts, including muscle activity and eye movement. The data are filtered using a zero phase band pass filters in the frequency band of 0.53∼60 Hz, (using MATLAB’s filtfilt function) (Hosseini et al., 2010).

### 2.4. Feature calculation

**Figure 2.**
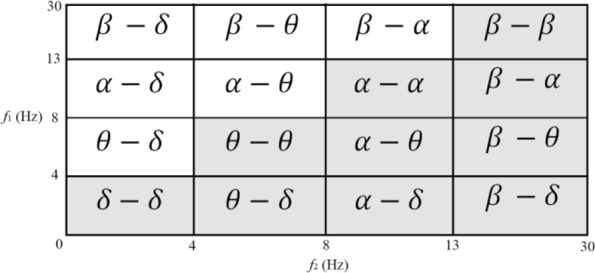
The different frequency ranges used for analysis in bi-frequency plane

The simulations are implemented in MATLAB and HOSA toolbox (Swami, Mendel, & Nikias, 2003). EEG segments are used corresponding to 23.6/11.8=2 seconds for feature extraction. The bispectrum is computed using direct-FFT and indirect methods. The bicoherence is also computed using the direct-FFT method. To define the features, I have 4 frequency intervals on each axis, leading to distinct regions as can be seen in Figure 2.

Four quantity indexes of “sum of the bispectrum magnitudes: ( Σ|Bis| )”, “sum of the squares of the bispectrum magnitudes: ( $\Sigma {\left|\text{Bis}\right|}^{2}$ )”, “sum of the bicoherence magnitudes: ( Σ|Bic| )”, and “sum of the squares of the bicoherence magnitudes: ( $\Sigma {\left|\text{Bic}\right|}^{2}$ )” in each of 10 regions and also in the whole frequency range are calculated (Overall 11 features). Eleven features and three features achieved from Hinich’s tests for Gaussian and linearity, including Chi-squared (χ^2^), Lambda (λ), and probability of false alarm (Pfa) add up to make 11×4+3=47 features for each EEG segment.

### 2.5. Feature normalization

Features are standardized to zero mean and unit SD by the following equation.

$$X_{i}^{\prime }=\frac{X_{i}-m}{\sigma },i=1,2,\ldots ,N.$$

Where N is the number of instances in a specific feature X, X_i_ and X′_i_ are the feature vectors prior and after the standardization, m and σ are the mean value and SD of each feature, respectively.

### 2.6. Feature selection

#### 2.6.1 Genetic algorithm

The feature space defined by the input signals contains overlapping features that should not affect classifier performance. Hence, an efficient approach is needed to identify and remove the feature redundancy. Here, the GA (Haupt & Haupt, 2004) is used for optimum features selection. GA strategy is summarized up in seven steps (Harikumar, Raghavan, & Sukanesh, 2005): 1. Prepare a randomly generated individuals of chromosomes (With binary encoding); 2. In each generation, calculate fitness of each chromosome; 3. Choose a pair of parent chromosomes from the initial population; 4. Choose a crossover probability rate (P_cross_=0.4), perform crossover to produce two offspring; 5. Mutate the two offspring with select a mutation probability rate (P_mutation_=0.05); 6. Replace the offspring in the population; and 7. Check for stopping criterion (a fixed number of generations, in here 100) or go to step 2.

The fitness of the chromosome is then updated based on the value of the classification accuracy of the trained network. Finally, optimum features are used as input to the LS-SVM with Gaussian and polynomial RBF kernels. The optimum features for the recognition of different epilepsy states in the first experiment are shown in Table 1.

**Table 1.** P-values for the experiment #1 including sets A, B, C, D, and E

**Row**	**Optimum Features**	**P**
1	$\Sigma {\left|{\text{Bis}}_{\alpha -\beta }\right|}^{2}$	8.64 E-10
2	$\Sigma {\left|{\text{Bis}}_{\delta -\delta }\right|}^{2}$	6.15 E-05
3	$\Sigma {\left|{\text{Bic}}_{\alpha -\beta }\right|}^{2}$	1.38 E-06
4	$\Sigma \left|{\text{Bic}}_{\text{whole}}\right|$	1.12 E-02
5	Pfa	1.78 E-04
6	$\Sigma {\left|{\text{Bis}}_{\alpha -\alpha }\right|}^{2}$	4.22 E-07
7	$\Sigma \left|{\text{Bis}}_{\theta -\delta }\right|$	0
8	$\Sigma \left|{\text{Bis}}_{\alpha -\delta }\right|$	0
9	λ	2.61 E-09
10	$\Sigma \left|{\text{Bis}}_{\beta -\beta }\right|$	7.21 E-03
11	$\Sigma {\left|{\text{Bis}}_{\beta -\theta }\right|}^{2}$	8.11 E-06
12	$\Sigma {\left|{\text{Bis}}_{\alpha -\alpha }\right|}^{2}$	0

Sets A and B are the healthy class, sets C and D are the interictal class, and set E is the ictal class.

#### 2.6.2 Feature ranking

The feature ranking method is used for identifying the best features for recognition of different epilepsy states. Here, the Student t test is used as the ranking method (Duda, Hart, & Stork, 2012). The analysis further confirms through the P values lower than 0.001 (i.e., with 99% confidence interval), to be used as best features for classification. The superior features in distinguishing the different classes in the experiment #1 using P value are shown in Table 1. As indicated here, $\Sigma {\left|{\text{Bis}}_{\alpha -\beta }\right|}^{2}$ , $\Sigma {\left|{\text{Bis}}_{\delta -\delta }\right|}^{2}$ , $\Sigma {\left|{\text{Bic}}_{\alpha -\beta }\right|}^{2}$ , $\Sigma \left|{\text{Bic}}_{\text{whole}}\right|$ , Pfa, $\Sigma {\left|{\text{Bic}}_{\alpha -\alpha }\right|}^{2}$ , λ, $\Sigma \left|{\text{Bis}}_{\beta -\beta }\right|$ and $\Sigma {\left|{\text{Bic}}_{\beta -\theta }\right|}^{2}$ provide better features.

### 2.7. Classification

A classifier utilizes diverse independent features as input to determine the corresponding class to which an independent feature belongs. The LS-SVMs were originally implemented for binary classification, but Suykens and Vandewalle (2002) proposed an extended version of LS-SVMs to multi-class problems, using different output coding methods such as Minimum Output Codes (MOC), Error Correcting Output Codes (ECOC), One-Versus-One (OVO), and One-Versus-All (OVA). Here, the multi-class LS-SVM is used with two different outputs coding methods of OVO and ECOC, using the Gaussian and polynomial RBF kernels. I also utilized the binary and multi-class LS-SVM, using LS-SVMlab toolbox (Suykens et al., 2002).

## 3. Results

To evaluate the ability of the proposed approach, we executed several experiments for the 5 sets of EEG signals. For the sake of comparison, 5 independent binary or multi-class classifiers were developed. The experiments were selected based on their clinical significance used in previous research studies. In the experiment #1, the EEG signals were classified into three classes; sets A and B were the healthy class, sets C and D the interictal class, and set E was the ictal class. In the experiment #2, sets, A, D and E classified into healthy, interictal, and ictal classes, respectively. In the experiment #3, sets A and E classified into healthy and ictal classes, respectively. In the experiment #4, sets A, B, C and D were combined together as the non-seizure class, whereas set E the seizure class. In the experiment #5, sets D and E classified into the interictal and ictal classes, respectively.

**Figure 3.**
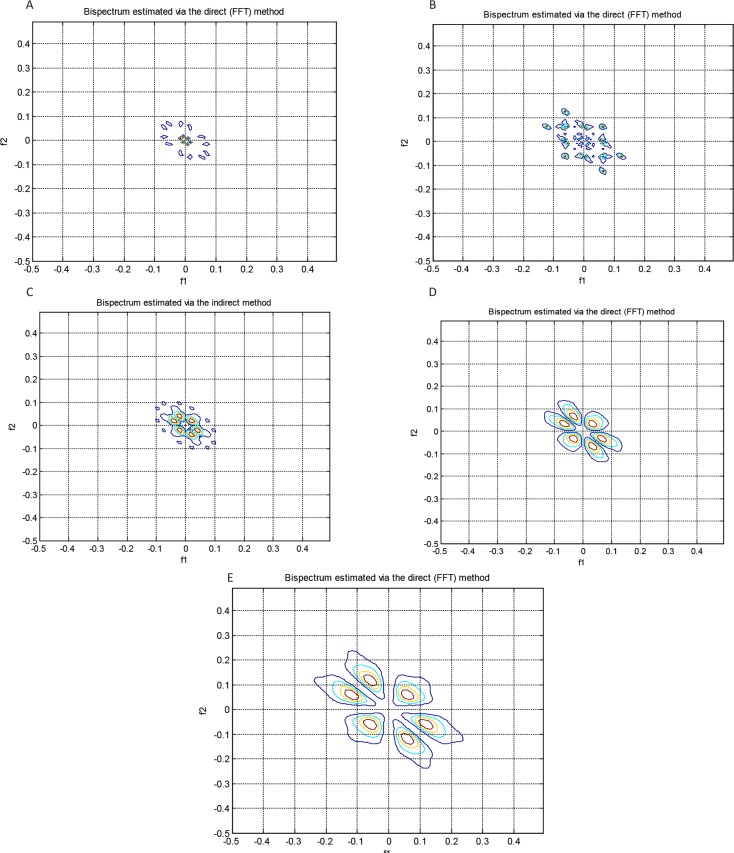
A contour plot of the magnitude of the direct estimated bispectrum on the bi-frequency plane, for a segment of datasets, “A”-“E”

**Figure 4.**
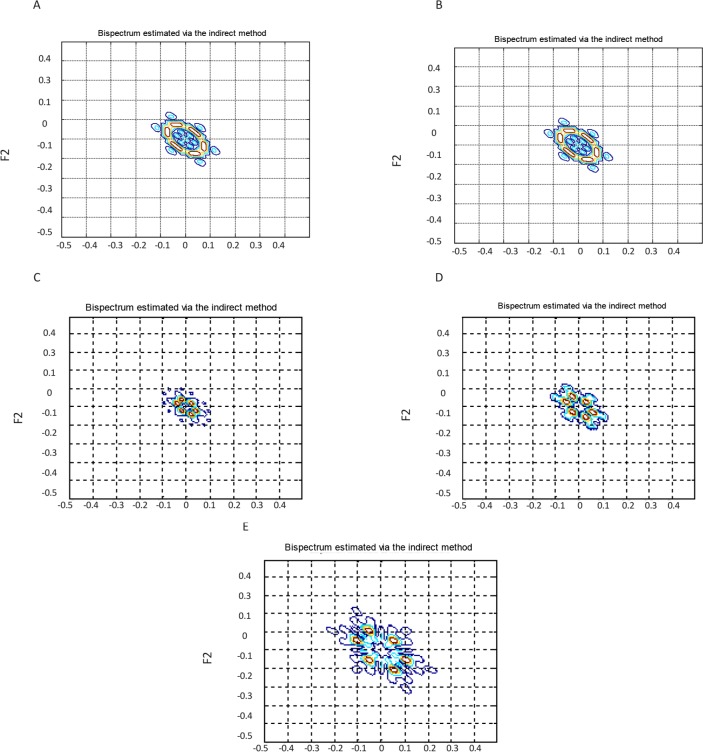
A contour plot of the magnitude of the indirect estimated bispectrum on the bi-frequency plane, for a segment of datasets, “A”-“E”

**Figure 5.**
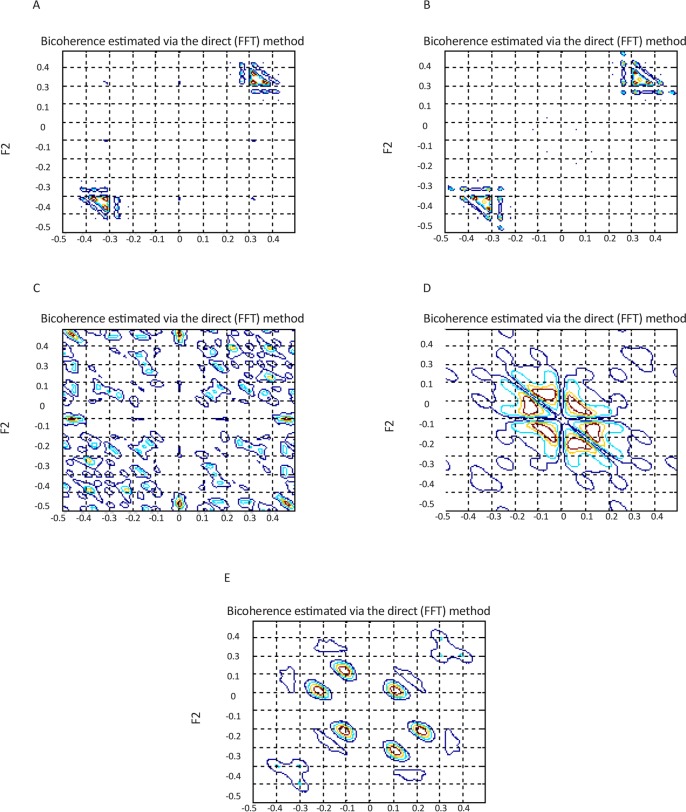
A contour plot of the magnitude of the direct estimated bicoherence on the bi-frequency plane, for a segment of datasets, “A”-“E”

After pre-processing, features of Σ|Bis| , $\Sigma {\left|\text{Bis}\right|}^{2}$ , Σ|Bic| , $\Sigma {\left|\text{Bic}\right|}^{2}$ , λ, χ^2^, and Pfa were extracted. The direct and indirect estimated bispectrum and direct estimated bicoherence on the bi-frequency plane are shown in Figures 3, 4, and 5. A contour plot of the magnitude of the direct estimated bispectrum on the bi-frequency plane is displayed in Figure 3. A contour plot of the magnitude of the indirect estimated bispectrum on the bi-frequency plane is displayed in Figure 4. A contour plot of the magnitude of the direct estimated bi-coherence on the bi-frequency plane is displayed in Figure 5.

The comparison of the plots shows a strong correlation between some two independent frequencies in non-linear systems. There are several bispectrum and bicoherence peaks in contour plots of bispectrum and bicoherence at every two bi-frequency plane, which reveals the location of the quadratic phase coupling. In this research, according to Table 2 around 60%, 35%, and 5% of the feature vectors were chosen randomly for training, testing, and validation, respectively.

**Table 2.** Distribution of the feature vectors randomly chosen for training, testing, and validation

**Experiments**		**Class**	**Number of Feature Vector Randomly Chosen**			

			**Training**	**Validation**	**Testing**	**Total**
#1		Healthy (A,B)	1920	160	1120	3200
		Interictal (C,D)	1920	160	1120	3200
		Ictal (E)	960	80	560	1600
	Total		4800	400	2800	8000
#2		Healthy (A)	960	80	560	1600
		Interictal (D)	960	80	560	1600
		Ictal (E)	960	80	560	1600
	Total		2880	240	1680	4800
#3		Healthy (A)	960	80	560	1600
		Ictal (E)	960	80	560	1600
	Total		1920	160	1120	3200
#4		Non-seizure (A,B,C,D)	3840	320	2240	6400
		Seizure (E)	960	80	560	1600
	Total		4800	400	2800	8000
#5		Interictal (D)	960	80	560	1600
		Ictal (E)	960	80	560	1600
	Total		1920	160	1120	3200

Additionally, maximum, minimum, and average fitness were calculated for the population of each GA. I used a set of statistical measures such as specificity, sensitivity, and total classification accuracy in order to compare our results with previous research. The performance of the classifier was determined by the statistical measures, which are defined as follows:

$$\begin{array}{l} \mathrm{Specificity}=\frac{T_{N}}{T_{N}+F_{P}}\times 100\%\\ \mathrm{Sensitivity}=\frac{T_{P}}{T_{P}+F_{N}}\times 100\%\\ \mathrm{Total}\;\mathrm{accuracy}=\frac{T_{P}+F_{N}}{T_{P}+F_{N}+F_{P}+F_{N}}\times 100\% \end{array}$$

The results of the binary or multi-class LS-SVM classifier with Gaussian and polynomial RBF kernels using two different output coding schemes are given in Tables 3 and 4.

**Table 3.** The results of the multi-class LS-SVM classifier with Gaussian and polynomial RBF kernels using two different output coding schemes

**Multi-Class LS-SVM Classifier**		**Without GA**				**With GA**			

		**Polynomial RBF**		**Gaussian RBF**		**Polynomial RBF**		**Gaussian RBF**	

		**OVO**	**ECOC**	**OVO**	**ECOC**	**OVO**	**ECOC**	**OVO**	**ECOC**
Experiment #1 (A,B), (C,D), E	Sensitivity AB	91.3	88.6	90.2	91.4	81.8	92.8	92.8	97.8
	Sensitivity CD	62.5	57.7	43.1	72.7	87.7	89.7	93.5	95.3
	Sensitivity E	87.8	71.7	80.1	84.3	91.6	98.2	94.1	100
	Total accuracy	79.1	72.9	69.3	82.5	86.1	92.6	93.3	97.2
Experiment #2 A, D, E	Sensitivity A	82.4	86.5	81.7	98.5	100	100	75.6	100
	Sensitivity D	76.7	66.7	74.1	94.1	97.5	96.7	94.3	100
	Sensitivity E	91.7	82.6	86.9	97.5	100	98.6	91.9	100
	Total accuracy	83.6	78.6	80.9	96.7	99.2	98.4	86.3	100

**Table 4.** The results of the binary LS-SVM classifier with polynomial and RBF kernels

**Binary LS-SVM Classifier**		**Without GA**		**With GA**	

		**Polynomial RBF**	**Gaussian RBF**	**Polynomial RBF**	**Gaussian RBF**
Experiment #3 A, E	Sensitivity	99.1	100	100	100
	Specificity	96.4	99.5	100	100
	Total accuracy	97.8	99.8	100	100
Experiment #4 (A, B, C, D), E	Sensitivity	91.9	95.1	100	100
	Specificity	90.6	90.4	100	99.8
	Total accuracy	91.3	92.8	100	99.9
Experiment #5 D, E	Sensitivity	91.1	94.4	99.6	100
	Specificity	89.8	91.6	96.1	100
	Total accuracy	90.5	93	97.9	100

## 4. Discussion

Table 3 shows that, in almost all the results, the combinations between GA and multi-class LS-SVM yield higher accuracy in comparison with not using GA. Thus, due to better results using GA, it is used to for reports in this section. For the experiment #1, the best result is obtained from ECOC LS-SVM with Gaussian RBF kernel, where the sensitivity for healthy, interictal, and ictal classes are 97.8%, 95.3%, and 100%, respectively. Also, the total accuracy has achieved 97.2%. For the experiment #2, the best result was obtained from ECOC LS-SVM with Gaussian RBF kernel, where the sensitivity for healthy, interictal, and ictal classes were all 100%, indicating a perfect classification of healthy, interictal, and ictal classes. Also, the total accuracy achieved 100%.

Also, Table 4 shows that, in almost all the results, the combinations of GA and LS-SVM yield higher accuracy in comparison with not using GA. Thus, due to the best results using GA, it is used to for reports in this section. For the experiment #3, the best result was obtained for LS-SVM with Gaussian and polynomial RBF kernels, where the sensitivity and specificity for healthy and ictal classes were all 100%. For the experiment is closely related to epilepsy diagnosis based on the presence of seizure activity only. For the experiment #4, the best result was obtained for LS-SVM with the polynomial RBF kernel, where the sensitivity and specificity for all of the data except ictal and ictal classes were 100%. For the experiment #5, the best result was obtained for LS-SVM with Gaussian RBF kernel, where the sensitivity and specificity for interictal and ictal classes were 100%. Finally, the best accuracies were obtained with the LS-SVM with Gaussian RBF kernel, except the experiment #4, in order to recognize different epilepsy states.

To compare performance against other competing algorithms, I performed a similar experimental procedure to theirs. Table 5 illustrates a comparison between the obtained results and previous studies results. For the experiment #1, the best results were obtained from Ktonas, (1987) and the proposed approach. For the experiment #2, the best results were achieved by Tzallas et al. (2009) and Alam & Bhuiyan (2013) and the proposed approach. For the experiment #3, the best results were obtained from Tzallas et al. (2009); Srinivasan et al., (2007) and Alam & Bhuiyan (2013) and the proposed approach. For the experiment #4, the best results were obtained from Alam & Bhuiyan (2013) and the proposed approach using LS-SVM with the polynomial RBF kernel.

**Table 5.** A comparison between the obtained results and previous studies

**Experiments**	**Reference**	**Methods**	**Accuracy (%)**
Expriment #1 (A, B), (C, D), E	(Tzallas, Tsipouras, & Fotiadis, 2007)	TFA and NN	97.72
	(Alam & Bhuiyan, 2013)	EMD, HOM, and NN	80
	The proposed approach	HOS, GA, and SVM	97.24
Expriment #2 A, D, E	(Tzallas, Tsipouras, & Fotiadis, 2007)	TFA and NN	99.28
	(Tzallas, Tsipouras, & Fotiadis, 2009)	RID and NN	100
	(Liang, Wang, & Chang, 2010)	TFA, ApEn, PCA, and SVM	∼98.67
	(Alam & Bhuiyan, 2013)	EMD, HOM, and NN	100
	The proposed approach	HOS, GA, and SVM	100
	(Subasi, 2007)	WCs and ANFIS	94
	(Tzallas, Tsipouras, & Fotiadis, 2007)	TFA and NN	100
	(Srinivasan, Eswaran, & Sriraam, 2007)	Entropy and Elman NN	100
	(Tzallas, Tsipouras, & Fotiadis, 2009)	RID and NN	100
	(Tezel & özbay, 2009)	NNAFF	∼100
	(Bedeeuzzaman, Farooq, & Khan, 2010)	Statistical distributions and Linear classifier	97.77
	(Guo et al., 2010)	WCs and NN	99.6
	(Fathima et al., 2011)	Statistical distributions and Linear classifier	96.9
	(Fu et al., 2015)	HMS and SVM	99.85
	(Daliri, 2013)	EMD and SVM	99.68
	(Alam & Bhuiyan, 2013)	EMD, HOM, and NN	100
	The proposed approach	HOS, GA, and SVM	100
Expriment #4 (A, B, C, D), E	(Tzallas, Tsipouras, & Fotiadis, 2007)	TFA and NN	97.73
	(Ocak, 2009)	WCs and Entropy	96.65
	(Liang, Wang, & Chang, 2010)	TFA, ApEn, and PCA	∼98.51
	(Guo et al., 2010)	WCs and NN	97.77
	(Alam & Bhuiyan, 2013)	EMD, HOM, and NN	100
	The proposed approach	HOS, GA, and SVM	99.9
Expriment #5 D, E	(Liang, Wang, & Chang, 2010)	TFA, ApEn, PCA, and SVM	98.74
	(Fu et al., 2015)	HMS and SVM	98.8
	(Alam & Bhuiyan, 2013)	EMD, HOM, and NN	100
	The proposed approach	HOS, GA, and SVM	100

For the experiment #5, the best results were achieved for Alam & Bhuiyan (2013) and the proposed approach. Also, it is noteworthy that, in spite of 100% accuracy of Alam & Bhuiyan (2013) in the experiments #2-#5, it did not provide satisfactory accuracy as in the experiment #1. We see that, except the experiment #1, the best accuracy was always obtained from Alam & Bhuiyan (2013) and the proposed approach, and, in the experiment #1 the accuracy of the proposed approach was very close (0.48%) to better accuracy, but far better (17.24%) than Alam & Bhuiyan (2013). The quantitative results show that the proposed approach can reach, in almost all of the experiments, up to 100% performance in terms of sensitivity, specificity, and accuracy.

This paper proposes a hybrid approach based on HOS for recognition of different epilepsy states using EEG signals. HOS provides valuable phase information that is not presented in the power spectrum. After pre-processing, HOS features such as bispectrum, bicoherence, and Hinich’s test are extracted from the EEG signals. Then, a GA is used to select optimum features. The analysis further confirms through the values lower than 0.001 for the extracting best features. These best features are used with a binary or multi-class LS-SVM with Gaussian and polynomial RBF kernels in order to recognize three different categories including healthy, interictal, and ictal states. The proposed approach is validated on a publicly available benchmark dataset in order to compare with previous studies.

The proposed approach is performed with bispectrum and bicoherence contour plots in both qualitative and quantitative perspectives. The qualitative results show several bispectrum and bicoherence peaks at every bi-frequency plane, which reveals the location of the quadratic phase coupling. An important contribution to the understanding of the dynamics of the epileptic brain may be found in Figures 3, 4, and 5. The quantitative results show that the proposed approach can reach, in almost all of the experiments, up to 100% performance in terms of, sensitivity, specificity, and accuracy. A final comparison between the obtained results and previous studies on the same database is presented to show the effectiveness of the proposed approach for seizure and epilepsy recognition.

Finally, HOS is an accurate tool in recognition of EEG signals in different epilepsy states. In the future, I intend to further validate the proposed approach with high density using larger clinical EEG databases. The striking feature for this study can be the morphological similarity of the plots of the different states (visual analysis of contour plots brings to mind the concepts of ‘self-similarity’ and through it, the connotation of fractals) which if confirmed, would yield unexpected and enlightening insights into the various states of the epileptic brain and their transitions (attractor deformation). Admittedly this is a speculation, but one that this reviewer regards as worthy of intense scrutiny.
